# Ultrasound localization microscopy

**DOI:** 10.1016/j.zemedi.2023.02.004

**Published:** 2023-06-15

**Authors:** Stefanie Dencks, Georg Schmitz

**Affiliations:** Lehrstuhl für Medizintechnik, Fakultät für Elektrotechnik und Informationstechnik, Ruhr-Universität Bochum, Bochum, Germany

**Keywords:** Super-resolution, Localization microscopy, Contrast agents, Microvasculature, Blood flow velocities, Tracking

## Abstract

Ultrasound Localization Microscopy (ULM) is an emerging technique that provides impressive super-resolved images of microvasculature, i.e., images with much better resolution than the conventional diffraction-limited ultrasound techniques and is already taking its first steps from preclinical to clinical applications. In comparison to the established perfusion or flow measurement methods, namely contrast-enhanced ultrasound (CEUS) and Doppler techniques, ULM allows imaging and flow measurements even down to the capillary level. As ULM can be realized as a post-processing method, conventional ultrasound systems can be used for.

ULM relies on the localization of single microbubbles (MB) of commercial, clinically approved contrast agents. In general, these very small and strong scatterers with typical radii of 1-3 *µm* are imaged much larger in ultrasound images than they actually are due to the point spread function of the imaging system. However, by applying appropriate methods, these MBs can be localized with sub-pixel precision. Then, by tracking MBs over successive frames of image sequences, not only the morphology of vascular trees but also functional information such as flow velocities or directions can be obtained and visualized. In addition, quantitative parameters can be derived to describe pathological and physiological changes in the microvasculature.

In this review, the general concept of ULM and conditions for its applicability to microvessel imaging are explained. Based on this, various aspects of the different processing steps for a concrete implementation are discussed. The trade-off between complete reconstruction of the microvasculature and the necessary measurement time as well as the implementation in 3D are reviewed in more detail, as they are the focus of current research. Through an overview of potential or already realized preclinical and clinical applications – pathologic angiogenesis or degeneration of vessels, physiological angiogenesis, or the general understanding of organ or tissue function – the great potential of ULM is demonstrated.

## 1 Introduction

**Figure 1:**
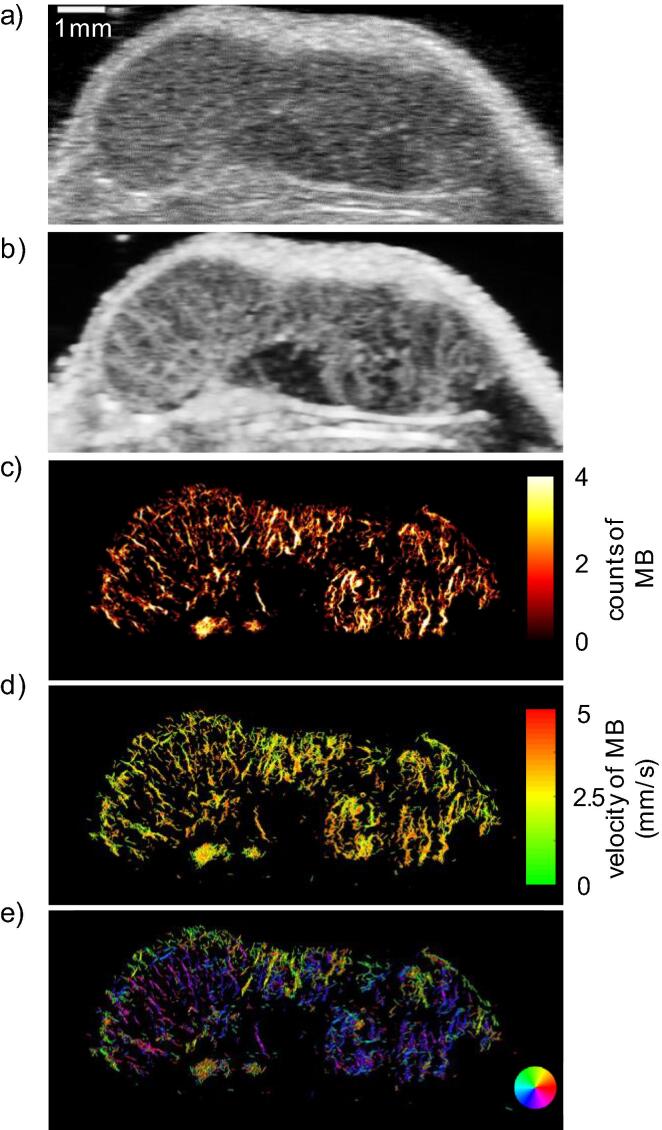
B-mode image of a murine xenograft tumor (a). Maximum intensity persistence image (b), ULM occurrences (c), ULM velocities (d), and ULM flow directions (e) in the tumor. Data were recorded at University Hospital Aachen with a Visualsonics Vevo 3100 at 18 MHz with transducer MX250S and MicroMarker contrast agent and processed with the ULM algorithms published in [7]. Approval of the animal experiment by the German State Office for Nature, Environment and Consumer Protection (LANUV) North Rhine-Westphalia.

More than a decade ago, sub-pixel localization of individual microbubbles in ultrasound images – later called ultrasound localization microscopy (ULM) – was demonstrated independently by Couture et al. [1] and by Siepmann et al. [2]. With this novel contrast-enhanced ultrasound imaging method vascular structures can be resolved below the diffraction limit of half-a-wavelength and typical resolutions in the range of a fifth to a tenth of the wavelength can be achieved. As ULM can be realized as a post-processing method, conventional ultrasound systems can be used for the acquisition. The contrast agents needed to apply the method, are clinically approved, and have been used for several decades in contrast-enhanced ultrasound (CEUS) imaging. They are administered intravenously during the acquisition. ULM then exploits the fact that ultrasound contrast agents consist of stabilized gas-filled microbubbles that scatter strongly and can be detected sensitively even as individual scatterers. While the image of a single microbubble (MB) is diffraction-limited with a maximum resolution of half-a-wavelength, the MBs can be localized with a much higher precision well below this limit [3]. By accumulating the microbubble localizations of imaging sequences in a high-resolution image, super-resolution vascular images can be generated (see Fig. 1-c; for comparison, a standard B-mode image is shown in Fig. 1-a). Additionally, when MBs are tracked along their path, functional parameters, such as flow velocities and directions of the MBs in the blood, can be derived and more complete vessel trees can be reconstructed (see Fig. 1-d and 1-e, respectively).

The underlying principle of localization microscopy was first established in optical imaging. Betzig et al. [4] proposed photo-activated localization microscopy (PALM), in which photoactivatable fluorescent protein molecules are used. The fluorescence of these molecules is stochastically activated at low densities and bleached after activation. By this, the light spots from single molecules are separated in consecutive excitations and their centers can be localized with high precision. As a similar technique stochastic optical reconstruction microscopy (STORM) was demonstrated [5], [6], in which the stochastic blinking of fluorescence signals over time was exploited to separate single molecule responses.

In comparison, ULM also uses the temporal variation of single MB signals because the MBs move with the blood flow and occur at different positions in consecutive frames. The motion of an MB in a vessel can be seen as deterministic since it can be calculated based on the blood flow velocity profile in this vessel. However, which vessels are flown through by the MBs is a stochastic process as the vessels are chosen randomly by the individual MB. It is important to make this distinction because the deterministic modelling can be used for tracking while the stochastic modelling is used to estimate the degree of reconstruction of the vasculature.

In the past, mainly two other ultrasound techniques have been established for vascular imaging and flow or perfusion measurements: CEUS and advanced Doppler techniques.

In CEUS the increased backscattering intensity of MBs in the images is directly used for diagnosis, either by qualitative observations of perfusion in the contrast-enhanced images or by quantitatively evaluating the maximum intensity over time in each pixel, which is called maximum-intensity-persistence (MIP, see an example in Fig. 1-b). For this, either an MB bolus is injected or the MBs are infused continuously and, e.g., evaluated with the destruction-replenishment method [8], in which all MBs in an imaging slice are destructed and the reperfusion is observed and evaluated. Quantitative perfusion parameters are then derived from time-intensity curves (TIC) as time-to-peak, peak-enhancement, and area-under-curve. One of the limitations of conventional CEUS is its high variability of the derived quantitative parameters due to factors relating to the scanner settings, to the patient, and to the MBs and their injection and dosage [9]. Moreover, as it is diffraction-limited in resolution, morphology down to the capillary level cannot be evaluated. Additionally, motion can disturb these quantitative measures by shifting tissues relative to the region of interest or out of the imaging plane.

**Figure 2:**
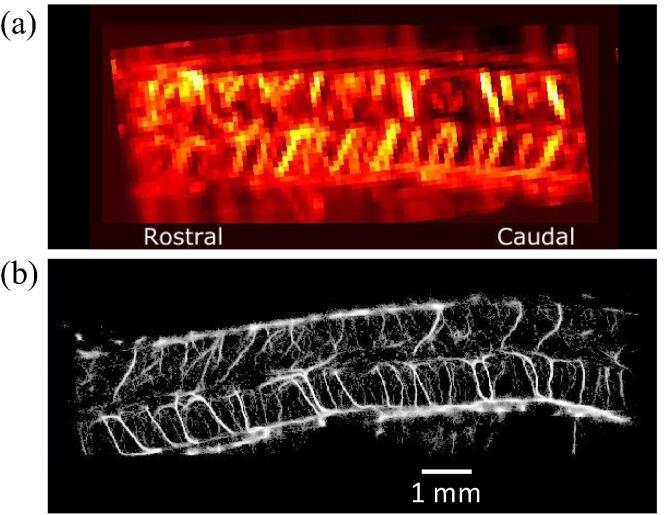
Ultrafast Doppler Imaging of the intact spine of a rat (a) and Ultrasound Localization Microscopy (b) in the same animal. Scale bar 1 mm for both images. Figure adapted from [11], creative commons license CC-BY-4.0, combined from Fig. 2A (intact) and Fig. 4B (intact) and scaled to identical scale.

While CEUS techniques are often used to derive quantitative parameters of tissue perfusion without resolving vessel details, Doppler techniques can be used to image blood flow in larger vessels. Using high frame rates in combination with plane wave imaging the sensitivity of Doppler was pushed towards imaging of smaller vessels. With a so-called µDoppler sequence based on the color Doppler technique combined with plane wave compound imaging, detailed images of, e.g., the rat brain [10] or the rat spinal cord [11] were generated. This technique was further improved to multi-angle vector Doppler imaging with which the angle-dependence of the velocity estimation was removed [12]. With these, user-independent functional parameters like flow velocities and directions can be evaluated. However, as these techniques are also diffraction-limited in resolution they can only be used for the evaluation of larger vessels or local perfusion. A comparison is shown in Fig. 2. The upper panel shows the sensitive ultrafast Doppler image in the rat’s spine in which vessels can be already identified. In the lower panel the superior resolution of the vasculature by ULM can be perceived.

Therefore, ULM imaging is complementary to existing methods in showing the microvasculature at unprecedented resolution. This enhanced resolution is also available over the full imaging depth that can be achieved with conventional ultrasound imaging, which opens many opportunities in diagnostic imaging and therapy monitoring for diseases that show alterations in the microvessels.

The remainder of this paper is organized as follows: First the basic concept of ULM is described. Different processing steps needed to generate the final ULM image and various algorithmic choices to be made – often depending on the application – are discussed in the second section. The third section highlights two current research foci in ULM. One concerns the stochastic aspect of the resulting images: Since the vessel tree is randomly sampled by the flowing MB, a certain amount of microvessels will not be sampled in a limited acquisition time. This poses questions about the degree of reconstruction of the vessel tree for a given measurement time and the definition of resolution, which are discussed here. A further research focus is the extension to three-dimensional ULM and eventually its translation to the clinic. In the last section clinical and preclinical applications and opportunities of ULM are discussed.

## 2 Basic concept

**Figure 3:**
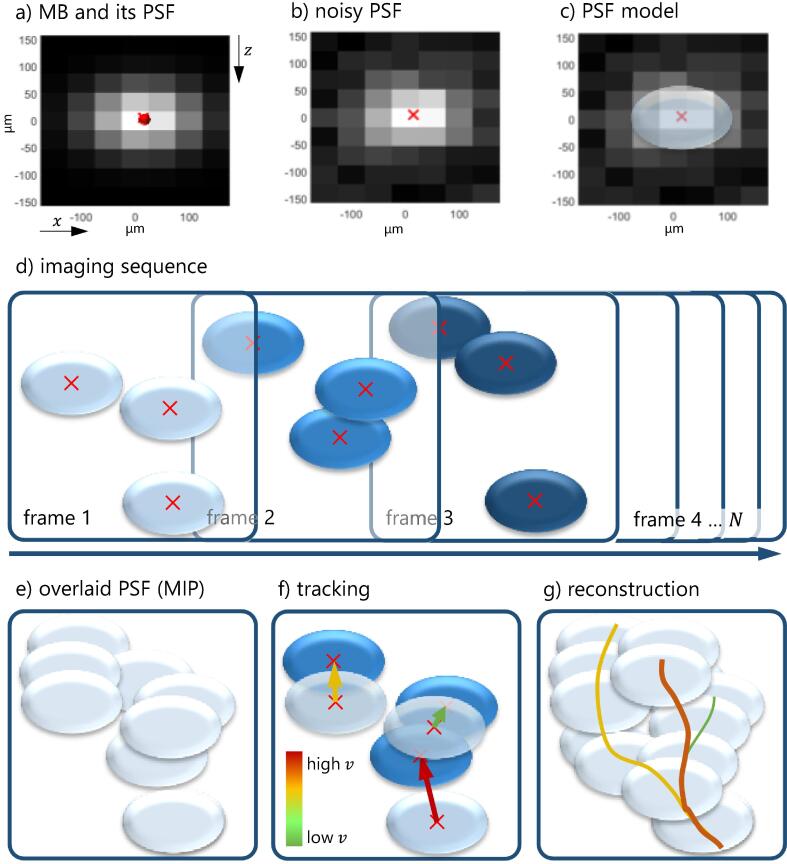
Basic concept of ULM. (a-c) Point spread function (PSF) of an MB in a B-mode image. The PSF is much larger than the original size of the MB (a: the MB is magnified and shown as red dot) and often disrupted by noise (b). The sub-pixel position of the MB (b and c: red cross) can be localized by, e.g., fitting a bivariate Gaussian function (see c) to the PSF. (d) Schematic illustration of the localization of single MBs in frame 1 to 3 of an imaging sequence. (e) *Maximum intensity projection* (MIP) of frame 1 to 3 using *contrast-enhanced ultrasound*. (f) Tracking of the MB’s positions from frame to frame (exemplarily shown for frame 1 to 2) allows the reconstruction of vessel courses and the determination of flow velocities and directions. (g) Reconstruction of the vessel tree from the full imaging sequence.

The basic concept of ULM relies on the presence of single isolated scatterers that can be detected sensitively in the image data and localized with high precision. Such scatterers are provided by ultrasound contrast agents, which consist of a large number of gas-filled microbubbles with typical radii of 1-3 µm. The MBs are encapsulated by a shell to increase their time in the blood pool to several minutes. Some commercial contrast agents for clinical or preclinical use are listed in Table1. These very small scatterers are imaged much larger in the ultrasound image due to spreading by the imaging system, which only has a diffraction-limited resolution, optimally reaching half-a-wavelength. This response of an imaging system to a point object is defined as the point spread function (PSF). In ultrasound systems the PSF’s axial extent, i.e., in direction of the sound propagation, is often about one wavelength. The lateral extent is larger, giving the PSF approximately an elliptical shape. In Fig. 3-a, a typical PSF is shown together with an MB visualized in red. Since the MB size of 1-3 µm is much smaller than the pixel dimensions here of Δx=50μm and Δz=35μm, the MB had to be drawn larger to be visible. However, it becomes clear that the position of the MB can be off-center in a pixel. Typically, the PSF is disrupted by noise, as shown in Fig. 3-b.

**Table 1:** Overview of several commercial ultrasound contrast agents for clinical and preclinical use. All contrast agents consist of microbubbles with diameters of several micrometers for which the composition (gas, shell) is listed according to the manufacturer’s information.

Name	Manufacturer	Shell	Gas	Mean / Median diameter [µm]
**Clinical**				

SonoVue/Lumason	Bracco Diagnostics	Phospholipid	Sulfurhexafluoride SF_6_	2-3 mean
Optison	GE Healthcare	Protein (human serum albumin)	Sulfurhexafluoride SF_6_	3-4.5 mean
Definity/Luminity	Lantheus Medical Imaging	Phospholipid	Perflutren/Octafluoropropane C_3_F_8_	1.1-2.5 mean
Sonazoid	GE Healthcare	Phosphatidylserine	Perfluorobutane C_4_F_10_	2.6 median

**Preclinical**				

MicromarkerUntargeted	Visualsonics / Bracco	Polyethylene glycol, Phospholipids, fatty acid	Perfluorobutane C_4_F_10_Nitrogen N_2_	2.3-2.9 median
SonoMAC-r	SonoMAC	Poly n-Butyl Cyanoacrylate	Air	1-2 mean

**Figure 4:**
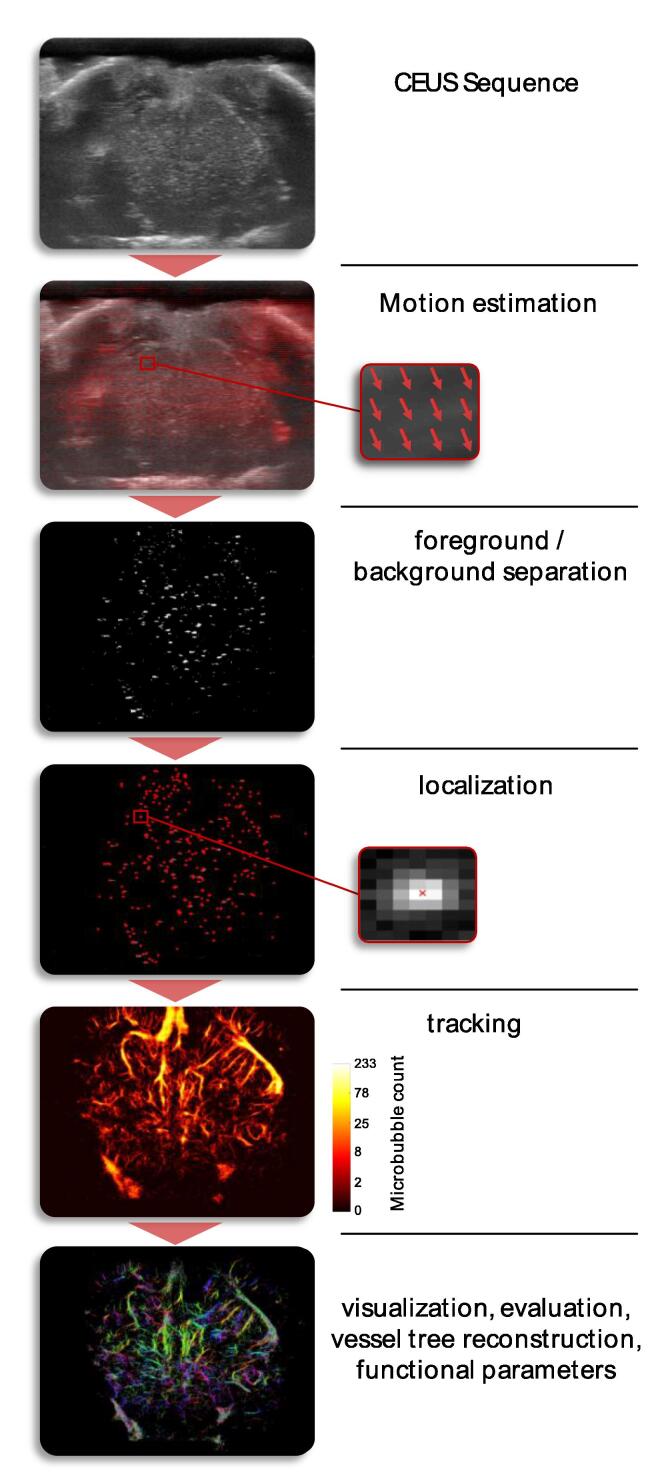
ULM processing steps. The example shown is a preclinical sequence of a mouse brain recorded with a MX700 transducer (29-71 MHz) on a Vevo 3100 (both Fujifilm Visualsonics). The contrast agent used for the CEUS sequence was MicroMarker (Fujifilm Visualsonics, see Table 1).

The resolution enhancement of ULM now relies on the fact that using appropriate methods, the MB position (red cross) can be estimated with a precision which is below the pixel dimensions and also much below the PSF size. Although other localization methods exist, the method that makes the concept of super-resolution easiest to understand is the least-squares fitting of a bivariate Gaussian function to the PSF (see Fig. 3-c).

If the PSF covered only one single pixel, super-resolution imaging would not be possible because the estimated position would always be the center of this pixel. For ULM, the PSF must cover multiple pixels, which allows the off-center position of the MB to be estimated with an accuracy higher than the pixel resolution. Therefore, the image pixel size must be smaller than the PSF extent. Additionally, signals from other MBs should not disturb the fit and therefore precise localization also requires spatially isolated PSFs, i.e., a certain sparseness of the MBs. The amount of overlap and the maximum MB density acceptable in the image depend on the exact localization method.

For ULM, the movement of MBs is observed over an imaging sequence (see Fig. 3-d). In each frame, the positions of the MBs are estimated. Computing the MIP of conventional CEUS, vessels are imaged much larger than they are, and a mainly bright image would be obtained (see Fig. 3-e, accumulation of frame 1 to 3). For ULM, the flow of the MBs is tracked by assigning localizations in one frame to the localizations in the next frame as shown in Fig. 3-f. This way, not only detailed morphological information on the course of the blood vessels but also functional information on flow velocities and directions are obtained. This information is gathered in maps of pixel dimensions much smaller (typically Δ=5-10μm) than the original pixel dimensions.

In principle, different visualizations of ULM are available: 1) For the occurrence map, the number of MB passing each pixel is counted. Either only the detections may be counted, or also inter-frame positions derived from the tracks can be used. Fig. 1-c shows an example of an occurrence map. 2) For the velocity map (see Fig. 3-g), the tracks of the MBs are colored according to the flow velocities estimated from the tracks (see example in Fig. 1-d). 3) In the direction map (see Fig. 1-e), the flow directions of the MB in the tracks are color coded. This is useful to distinguish closely spaced blood vessels of opposite flow direction or understand the inflow and outflow perfusion pattern of the microvasculature. 4) Additionally, the time points of detections can be color coded. This way, local perfusion abnormalities can be made visible. Regardless of the choice of visualization, it becomes clear that the reconstruction of the vasculature is substantially more detailed than with CEUS.

**Figure 5:**
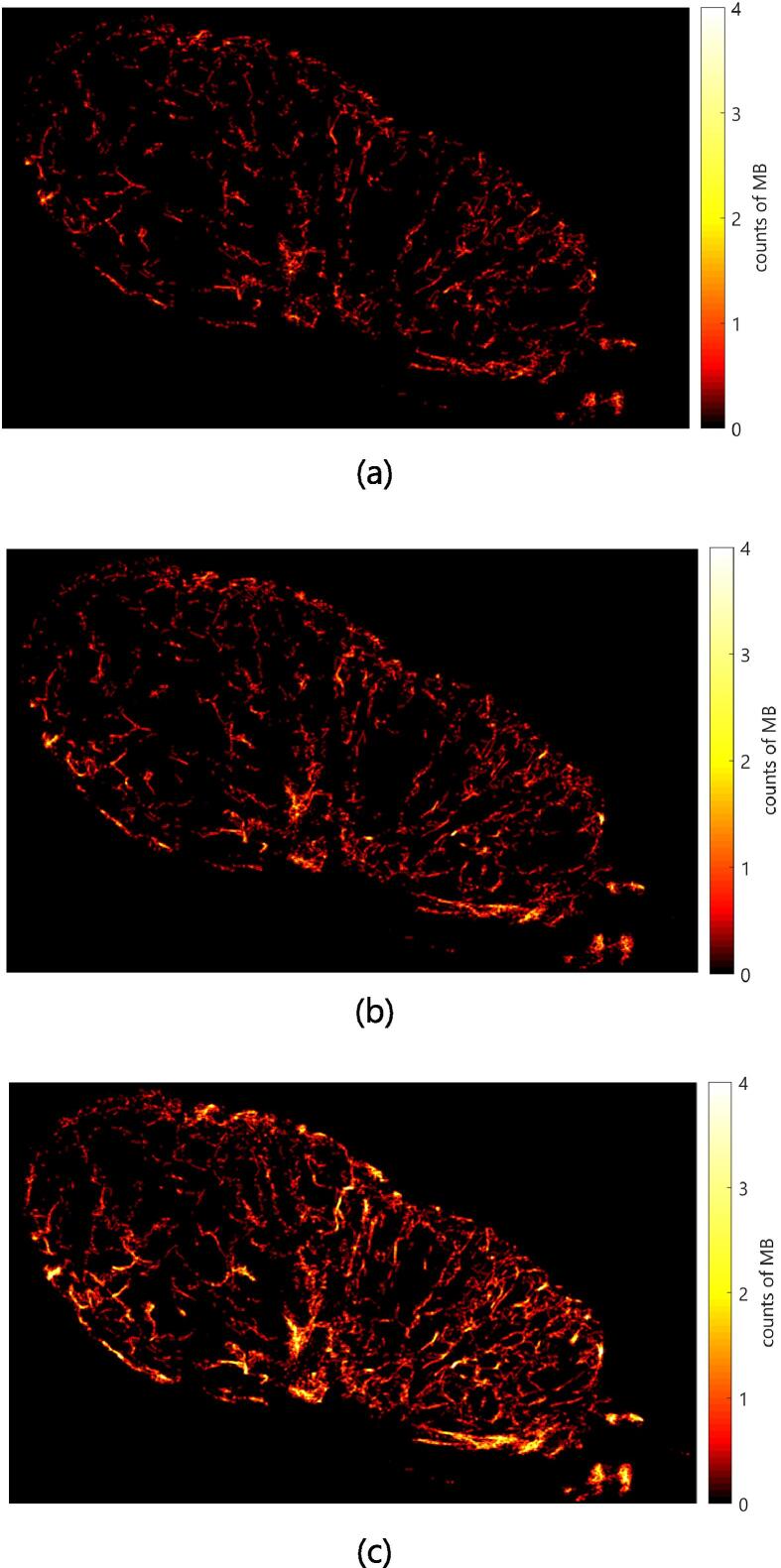
Reconstruction of the microvasculature of an A431 murine xenograft tumor (data set 2, see Section IV results) after (a) 10, (b) 20, and (c) 40 s. From [22] under Creative Commons License CC-BY-4.0.

Based on these reconstructions, novel quantitative measures of the microvascular morphology and function can be derived. For example, the relative blood volume (rBV) can be estimated from the ratio between pixels that contain tracks and all pixels. Other parameters are derived from statistical evaluations of the vessel length and flow velocity distributions. Especially for measurements on tumors, the quantitative characterization of vascular branching or tortuosity are of interest as it is known that the immature vasculature of tumors shows more frequent branching and a higher tortuosity of vessels [13], [14]. An important aspect of quantitative parameters derived from ULM is their possible dependence on the acquisition time and the concentration of MBs because they influence the random sampling and completeness of the vessel tree. This is demonstrated in Fig. 5, where vessel tracks are accumulated over 10 s, 20 s, and 40 s, showing increasing detail and vessel density. Thus, quantitative parameters must be carefully normalized to become independent of variations in acquisition conditions.

## 3 ULM processing steps

Generally, ULM can be realized in different ways and acquisitions from preclinical and clinical systems can be used. Most clinical ultrasound scanners still scan the image area in a line-by-line fashion with focused transmissions and have typical frame rates in the range of 5-50Hz. Some newer systems and research scanners also realize unfocused transmissions of plane or diverging waves that allow to reconstruct images with a trade-off between imaging framerate and image quality and can reach frame rates of several kHz [15]. While some works see these high frame rates as an essential component of ULM, high resolution images can also be generated with lower frame rates but may need more sophisticated tracking algorithms depending on the application. For example, the ULM image in Fig. 1 was acquired with line-by-line scanning at a frame rate of 50 Hz, while the ULM image in Fig. 2 was acquired with plane waves and a frame rate of 1 kHz. Whether an ultrasound system together with an acquisition protocol is suitable for ULM depends on the specific application and the algorithm used to reconstruct the vessel images. The different aspects that must be considered are discussed in the following.

### 3.1 Type of image data

For many of the processing steps described in the following subsections, the type of image data that are available to reconstruct ULM images has an influence on the choice of algorithms and their performance. Ultrasound systems transmit a short pulse oscillating in the MHz-range. The received echo signal is the linear superposition of the signals from the tissue and MB scatterers. Such signals are referred to as rf-signals. In a first processing step the high-frequency rf-signals are shifted to the baseband resulting in the complex IQ-signal. Its real part is the in-phase component I and the imaginary part the quadrature component Q. The original rf-signal can be reconstructed from this IQ signal by up-mixing and thus IQ-signals still carry the complete information about the linear superposition of the scatterers. In a last step, the magnitude of the complex IQ signals is calculated to detect the envelope of the rf-signals. The result is log-compressed and converted to the gray-values shown on the conventional B-mode image. In the nonlinear processing step of envelope detection, the phase information and the linear superposition of the scatterers’ signals is lost. Furthermore, the exact log-compression and gray-value transformation is often unknown for commercial scanners. Therefore, B-mode or envelope data does not allow to linearly decompose the signals anymore which has consequences for some processing steps. However, most clinical scanners only provide such B-mode data via their DICOM interface and though processing of B-mode data is suboptimal, it is often the only viable path to early clinical application of ULM before its implementation in clinical scanners.

### 3.2 US imaging of MB

From conventional ultrasound imaging it is known that higher US frequencies allow for a better resolution but limit the imaging depth due to their higher attenuation. While the limitation of the imaging depth continues to exist also for ULM, the direct dependence of the resolution on the frequency is not valid anymore without considering the spatial sampling of the PSF and the signal-to-noise ratio (SNR) of the images. In a recent study, we could show that using the lower frequency US probe of a clinical US scanner a better localization precision could be achieved than using the higher frequency probe when the DICOM images as provided by the manufacturer were used [16]. Nevertheless, the choice of the US probe and, thus, the frequency influences the visibility of the MBs. E.g., for high frequencies of 20-70MHz as used in preclinical imaging, hard-shelled (SonoMAC-r, SonoMAC) and soft-shelled (MicroMarker, FujiFilm Visualsonics) MBs are easily visible with a high signal-to-background ratio in the B-mode images. For these sequences, it is easy to detect and localize the MBs by image processing without the need for dedicated contrast-enhanced imaging modes. For the lower frequencies of clinical imaging of f≈2-18MHz scattering of the MBs is significantly reduced as their scattering coefficient is proportional to $f^{4}$. Superimposed to this general frequency dependence, the MBs show a resonance in the lower clinical frequency range. Still, in conventional B-mode images single MB often remain invisible. Therefore, the nonlinear response of the oscillating MB is exploited: Various dedicated contrast imaging modes have been developed and are available on clinical ultrasound scanners. These modes mostly work with multiple acquisitions with different transmit pulses to identify scatterers that have a nonlinear response. E.g., in the pulse inversion method [17], [18] two acquisitions of the same image line are performed with an excitation pulse that is inverted for the second transmission. If the echo responses to the two transmissions come from a linear scatterer they cancel while echoes from nonlinear scatterers result in a remaining signal. While MB signals are significantly enhanced by contrast-specific imaging, also tissue nonlinearities and signal saturation contribute to some background clutter that must be removed.

### 3.3 MB concentration

In addition to the choice of transducer and imaging modality, the MB concentration in the blood plays a substantial role for localization precision. Generally, the more MBs are localized the better the vasculature can be reconstructed. However, ULM relies on the localization of single MBs. If the MB concentration is too high, their PSFs are superimposed, and it becomes difficult to accurately localize their positions. Therefore, the injection protocol of the MB suspension has to be chosen carefully and often be optimized for a certain application in a first clinical dose-finding study. Generally, continuous infusion is preferred over a bolus injection. E.g., in a preclinical setting for imaging a mouse ear an infusion rate of 0.2-5.0μL/min of a SonoVue suspension at 2.5 times the original concentration of original vial is reported [19]. In clinical settings for imaging the microvasculature of a human lower limb and of tumors, 4mL/min of a vial of SonoVue diluted in saline [20] and 0.5mL SonoVue over 10s [21] were used, respectively.

### 3.4 Motion estimation and compensation

To sample the microvasculature with ULM in a two-dimensional image, recording times in the order of a minute up to 10 minutes have been reported by Dencks et al. [21], [22] and Errico et al. [23], respectively. One of the most critical aspects to achieve super-resolution is the handling of tissue motion. If not correctly compensated for, tissue motion leads to a loss of resolution by motion blur and can result in misleading reconstructions [20]. While this aspect can be neglected in measurements for which motion is suppressed, e.g., with a fixed US probe on the brain with a stereotactic fixation of the skull [23], a non-rigid motion compensation, e.g., of the kidney with strong superimposed breathing motion, is a challenge.

For two-dimensional imaging, motion can be within the image plane or perpendicular to it. In-plane motion can be estimated and compensated. Motion estimation algorithms from image processing have been used to estimate rigid motion [24], [25], a combination of affine and nonrigid motion [20], or complete motion vector fields, e.g., with optical flow [26]. In principle, motion estimation methods used in other US imaging techniques could also bring further improvements, e.g., regularized block matching for strain imaging [27] or convolutional neural networks for elastography. That also a 3D motion correction for volumetric ULM is feasible using the two-stage motion correction proposed in [20] has already been shown [28]. With the estimated motion, it is preferred to correct only the MB localizations and not to compensate the motion in the images before localization to avoid any deformation of the PSF. In contrast, out-of-plane motion cannot be compensated for, and three-dimensional imaging is needed for applications where this motion is dominant. In two-dimensional imaging, frames with strong out-of-plane motion can be detected by normalized cross-correlation and excluded from the analysis [2], [21], [25].

A problem for motion compensation in clinical imaging is due to the fact that the MBs can only be detected in contrast mode imaging. As these images suppress the tissue background, they are unsuitable for motion estimation and B-mode images must be acquired interleaved with contrast mode images further reducing the frame rate on clinical scanners.

### 3.5 Foreground-background separation

Independent of the imaging mode, a separation of the moving MB foreground from the stationary background must be carried out before localizing the MBs in the foreground images. For this, different methods can be used, e.g. frame-to-frame subtraction [3], [29] (or variations like subtracting frames with a time difference of three frames [2], a rolling background average [19], [30], or temporal rank filters [31] depending on the assumption about the relation between flow velocity and frame rate), singular value decomposition (SVD) [32], or a spatiotemporal nonlocal means (NLM) denoising filtering [33]. The performance of the different algorithms depends on the image data and the application and can have considerable influence on the localization precision when the shape of the expected PSF is deformed by an imperfect foreground-background separation. Some approaches assume a linear superposition of the foreground and the background as, e.g., the SVD method, and therefore need rf-data or IQ-data, in which these assumptions hold.

### 3.6 Localization

To localize single MB in the images, most algorithms first detect the MBs as objects in the foreground images and only small patches containing the PSF are processed. Various methods have been proposed for the accurate and precise localization of MBs from the noisy PSF patches. For example, maxima of the correlation with Gaussian PSF models on interpolated grids [33], the weighted average/center of mass [30], [34], model fits of the PSF with a Gaussian function [7], [23], matching of hyperbolas on pre-beamformed channel data [1], or radial symmetry [35] have been used. Which localization method is best depending on, e.g., their precision, sensitivity, robustness, or on the effects of MB concentration, pixel sizes or SNR is still under discussion [36], [37], [38], [39] and benchmarking by theoretical considerations or simulations depends on the exact algorithmic realization and the noise model that is chosen.

Recently, deep learning methods have shown to be more robust in case of high MB concentrations [39]. In these methods the mapping of the centers of several MBs from coarse patches to a finer grid is learned from simulated ground-truth examples. Another approach to handle high MB concentrations is the use of velocity filters [40], [41]. These are applied to suppress the PSF of MBs that do not belong to a respective flow velocity and direction defined by a filter bank. This way, the apparent MB density is reduced in each filtered image facilitating the localization. Afterwards the localizations of all filtered images are combined.

### 3.7 Tracking

While the accumulation of MB localizations could already show the microvasculature in super-resolution, it is beneficial to track the MBs over consecutive frames to fill intermediate positions and to estimate their flow direction and velocity. In most of the approaches the MBs are assumed to be indistinguishable, so that the association of MBs to tracks is based on model assumptions of their motion and select the most probable association. The easiest tracking method is nearest-neighbor tracking [23], in which the closest MB in the next frame is connected to continue a track. Because an MB at the same position in the next frame is the best match, it can be interpreted to have the underlying model assumption that the MB does not move from one frame to the next. Therefore, it is only a valid method for very high frame rates like 500 Hz as in [23], where motion from one frame to the next is minimized or for very low MB concentrations with no ambiguity in assignments. Nearest-neighbor assignment must be stopped from connecting far-away objects by limiting the allowed maximum distance. To resolve double-assignments, often bipartite graph matching algorithms as the Kuhn-Munkres or Hungarian algorithm are used [42], which minimize the distances jointly over all pairs – so-called balanced assignment problems. The bipartite graph-based microbubble tracking [33] is based on this algorithm taking into account that some detections “cannot or (should not) be paired” – a so-called unbalanced assignment problem. The James Munkres’s variant which is provided by the Matlab function assignDetectionsToTracks.m can also handle missing or false detection by defining costs for unassigned tracks or detections. As these methods also minimize the distance of positions between frames, they have the same implicit assumption as the nearest-neighbor method, which is a zero-velocity motion model: The optimal position for pairing an MB in the next frame is the position of a resting MB. This assumption only holds either for very small velocities or for very high frame rates.

If the frame rate is low (e.g., in a clinical US scanner around 15Hz [21]), the distances traveled between two acquisitions are large and if there are many MBs the assignments become ambiguous. This assignment problem becomes more difficult the more complex and denser the vasculature is. In 2D ULM, due to the projection of vessels into one plane because of the elevational slice thickness, apparent crossings of vessels may occur. To ensure reliable tracking even under such difficult conditions, motion models, often incorporated in Kalman filters of the MB positions, can be used. The Markov Chain Monte Carlo Data Association (MCMCDA) [43] adapted from radar tracking of multiple targets to ULM [34], [44] optimizes the overall probability of an association with a linear constant velocity Kalman filter motion model. Later Kalman filters were used to filter MB positions after tracking with the Munkres algorithm [45], however, the Kalman filter was not used for the association task. A hierarchical approach with different velocity ranges was described in [46] to associate tracks with a high variation in velocities. Solomon et al. [47] used a Kalman filter in combination with multiple hypothesis tracking (MHT), a multiple target tracking algorithm with an optimization of the overall assignment probability similar to MCMCDA. The optimization of tracking algorithms for ULM, their model assumptions and choice of parameters is still a very active field of research and motion models adapted to the actual flow in microvessels may lead to further improvements.

Up to here, the MBs were considered to be indistinguishable. If we assume that the MBs act as point-sources and the PSF of the imaging system is space-invariant this assumption is initially reasonable. However, the brightness and appearance of the MBs may vary due to their elevational position (in 2D ultrasound imaging) or due to their non-linear scattering. With simulations and *in-vitro* experiments it has been shown that non-linear scattering may cause an elongation and alteration of the original waveform and of the PSF in axial direction depending on the MB size [36]. While no tracking was performed in this study, in [19] only MBs were allowed to be assigned to each other for tracking if their cross-correlation was greater than 0.8 in the B-mode images.

Overall, linear programming, which assigns weights to the associations, would easily allow to integrate characteristics of MBs. E.g., in optical applications the particle intensity was considered for tracking [48]. The algorithm implemented there, was also used for ULM (then called uTrack [49]), but apparently without using this extension. Going one step further, not only image-intensity based features were integrated into the cost function but also their position probabilities (one time using a Kalman filter [50], one time forcing the MB movement to be small by a constraint [51]). Generally, different nonlinear responses would be more pronounced in rf-signals, because changed frequency components and phase jumps in case of resonance are detectable whereas this information will be largely lost in B-mode data.

Recently, two novel approaches were proposed: One combines the localization and tracking with a fully dynamical inversion scheme, i.e., “locations and velocities are simultaneously reconstructed” [52]. Since this approach was evaluated on simulation data in which a symmetric 2D Gaussian function was assumed to be the PSF, the improvements are yet to be investigated on real US data. For the other approach a deep learning neural network was implemented to realize super-resolution microbubble velocimetry without prior localization [53]. Here, the classical U-net structure [54] for extracting spatial features is combined with a long-short-term-memory-block for the temporal feature extraction. With this, a better spatial and temporal resolution than with conventional Doppler techniques were achieved providing even information on the flow pulsatility within the vessels. The resolution limits compared to ULM have not yet been reviewed.

### 3.8 Precision and resolution

The resolution achievable, i.e., the possibility to distinguish close vessels, is particularly limited by the precision of the MB localization. This precision is mainly dependent on the precision of measuring the MB signal’s time delay, but also on the focusing. For localization using rf-signals the time delay precision increases for a higher center frequency and larger pulse bandwidth, for a better SNR, and for a higher number of transducer elements used in the aperture (theoretically derived in [3]). For the lateral precision, additionally a larger aperture is advantageous. When working on B-mode data, also the localization method [36], [37], [38], [39] and the ratio between the PSF size and the pixel size is relevant [37]. As mentioned before, off-center positions of the MBs can only be estimated if the pixel size is smaller than the PSF extent. However, the final resolution of the ULM image will also depend on other factors than localization precision, e.g., MB concentration or the quality of motion correction.

For visualization, the tracks of the MB are drawn into maps of pixel dimensions smaller than the original B-mode image and typically around Δ=5-10μm. However, this pixel dimension does not correspond to the actual resolution of the image. In fact, it is not trivial to determine the resolution of *in-vivo* measurements without ground truth. First approaches consisted of exemplarily evaluating flow profiles of very close vessels [19], [23], a newer approach is the use of the *Fourier ring correlation* [55]. Both approaches evaluate the final image resolution also incorporating the effect of, e.g., motion or imaging artefacts in addition to localization precision. With the first approach, e.g., a resolution under 20μm ([19]: frame rate 25Hz, transducer frequency 6.5MHz, half-wavelength 118 *µ*m) and the possibility to distinguish vessels that are 20μm apart ([23]: frame rate 500Hz, transducer frequency 20.3MHz, half-wavelength 38 *µ*m) were reported. With the second approach, a resolution of 9-34μm was determined ([55]: frame rate 500-1000Hz, transducer frequency of 15MHz, half-wavelength 51 *µ*m).

## 4 Current research directions

### 4.1 Measurement time and degree of reconstruction

One of the main concerns for clinical application of ULM are the measurement times to acquire a reasonably filled microvessel image. To obtain a complete reconstruction of the microvasculature, each single capillary would have to be perfused at least once by an MB. The problem is not so much the dissolution of the MBs in the blood, as these are stable for a comparatively long time (several minutes) or even longer for constant infusion, but the limited measurement times that are tolerable for the patients. Thus, a compromise has to be found between the measurement times and the degree of reconstruction. For preclinical measurements at murine tumors, we reported a degree of reconstruction between 50%-70% after 40s, and for clinical tumor measurements about 40% after 47s and 75% after 90s [21]. Hingot et al. expect about 10min for a complete (nearly 100%) capillary reconstruction of a rat’s brain [56].

To establish suitable measurement protocols, it is important to be able to estimate the degree of reconstruction. It is also essential for the normalization of derived quantitative parameters, e.g., the calculated rBV value will be lower for lower reconstruction degrees. In first approaches, an exponential saturation model was fit to the number of filled pixels to all pixels in the super-resolved image over time or rather over the number of detected MBs over time from which the reconstruction degree could be derived [56], [58], [59]. Then, based on a relatively complex derivation, however, a much easier method was proposed: Assuming that the exact path an MB takes is chosen randomly, the filling of the vessel tree can be modeled as a spatiotemporal random process. Under the assumption that pixels belonging to vessels are visited by MBs randomly but with a fixed rate, it can be modelled as a Poisson process [21], [59], [60]. This model has the limitation that pixels that do not contain vessels will not be filled independent of the rate leading to an excess of pixels with zero counts. However, taking this into account by using a zero-inflated Poisson model allows to estimate the filling rate, the degree of reconstruction of the vessel tree, and the number of pixels that would eventually be filled with direct expressions that simply use the number of filled pixels and the total number of MB observations at the corresponding time point [22]. In Fig. 7, the estimated vessel probability ${\hat{P}}_{v}$ (final percentage of pixels containing vessels), which is equivalent to rBV, using this method is shown over the number of frames for a clinical dataset [22]. It can be seen, that after a certain time (around 700 evaluated frames) a nearly robust estimate of the vessel probability ${\hat{P}}_{v}$ of about 20% is achieved, although at this time point the filling of the image (percentage of pixels containing vessels named coverage C) had only reached about 8%, which corresponds to a degree of reconstruction of 40%.

### 4.2 3D ULM

One of the essential steps for the further development of ULM will be the full implementation of the method with fast three-dimensional ultrasound imaging. Several limitations of the method can be addressed in this way:

1) In the two-dimensional ULM, the MBs detected in a relatively thick slice are projected into the slice, making their separation for localization more difficult. Thus, in 3D higher concentrations could be allowed, which speeds up the acquisition of ULM images. Additionally, MBs can be imaged and localized in a whole volume, making optimal use of the available MBs during their circulation time. Obviously, localization and super-resolution is added in the third dimension, but additionally, the localization will be more precise because of the larger number of voxels of the 3D PSF, which reduces the estimation variance.

2) In 2D, the MB tracks are also projected into the image plane and thus velocities are often underestimated while in 3D the velocity vectors can be determined correctly.

3) Microbubbles can be tracked in a volume longer along continuous tracks, resulting in contiguous vessel trees.

4) Out-of-plane tissue motion is present in most clinical applications and can only be estimated and compensated in 3D. For clinical translation this last aspect is maybe the most important one.

However, the described advantages of 3D ULM can only be achieved with high volume rate imaging. This excludes building volumes from 2D ULM using slice-by-slice mechanical scanning as was done in first preclinical 3D studies: E.g., Zhu et al. [61] imaged 1.7 mm thick volume slices of lymph nodes of New Zealand white rabbits with a mechanically-scanned high-frequency linear array that rested 2.4 s in each of 17 slices with 40.8 s total acquisition time. Lin et al. [62] imaged tumor angiogenesis in rats with a mechanically scanned linear array at 4.5 MHz. Recording times for volumes were kept below 35 minutes by limiting the number of recorded slices. Özdemir et al. [63] used mechanical scanning of chicken-embryos with a 21 MHz probe and applied ULM without tracking. In these studies, the recorded volumes were still diffraction-limited in the mechanical scanning direction and out-of-plane tracking, or motion compensation was not possible. Christensen-Jeffries et al. [64] realized a fast volumetric acquisition using two linear arrays with their imaging planes intersecting at 90°. While they were able to show super-resolution for cellulose tubes that lied within the intersection volume, the approach has a very small field of view and would be difficult to be applied clinically.

Acquisitions with high volume rates have to use electronically steered matrix arrays. While such transducers are available in several clinical systems, the acquisition modes are often aimed at different applications – typically fast cardiac imaging – and via DICOM only volume data that has undergone many unknown processing steps is available. Christensen-Jeffries et al. have shown first results using data from a clinical matrix probe [65]. Such commercial matrix transducers often have several thousand transducer elements and use microbeamformers and preprocessing integrated into the probe. Because the raw data of such systems are not available and processing of data is suboptimal for ULM, researchers mainly turn to matrix transducers that are available for research scanners and cannot be used in clinical studies without further safety measurements and ethical clearance. Such a commercially available system used by several recent studies [42], [57], [66], [67], [68] is the probe from Vermon (Tours, France) that is, e.g., compatible with the Verasonics Vantage research scanner (Verasonics, Kirkland WA, USA). This transducer has a matrix of 35 × 32 transducer elements with three unconnected rows (9,18,27) resulting in 32 × 32 active elements, which need 1024 channels or multiple acquisitions with multiplexing.

Since the processing of the data of 1024 channels is time-consuming, some approaches have been proposed to record 3D data with a reduced channel number. Harput et al. [68] used the mentioned Vermon array with a sparse selection of elements and imaged a twisted pair of 200 µm diameter cellulose tubes with super-resolution. Still two 256 channel systems had to be used and the amount of recorded data is large. A different approach was proposed by Jensen et al. [69], who devised a row-column array, which uses two perpendicular linear arrays of 62 long elements (24.84 mm length) each for transmit and receive. Super-resolution was demonstrated in phantoms with volume rates of 156 Hz and the lower number of channels together with their larger apertures make row-column arrays interesting for clinical application of the method. The row-column array used by Jensen et al. [69] has an aperture of ca. 25 mm × 25 mm and thus achieves better focusing than the smaller Vermon 35 × 32 array (ca. 10 mm × 10 mm). However, 3D ULM is also possible with the 35 × 32 Vermon array using all transducer elements as demonstrated *in-vitro* by Heiles et al. [42] imaging a bifurcation in a vessel phantom with a volume rate of 500 MHz.

**Figure 6:**
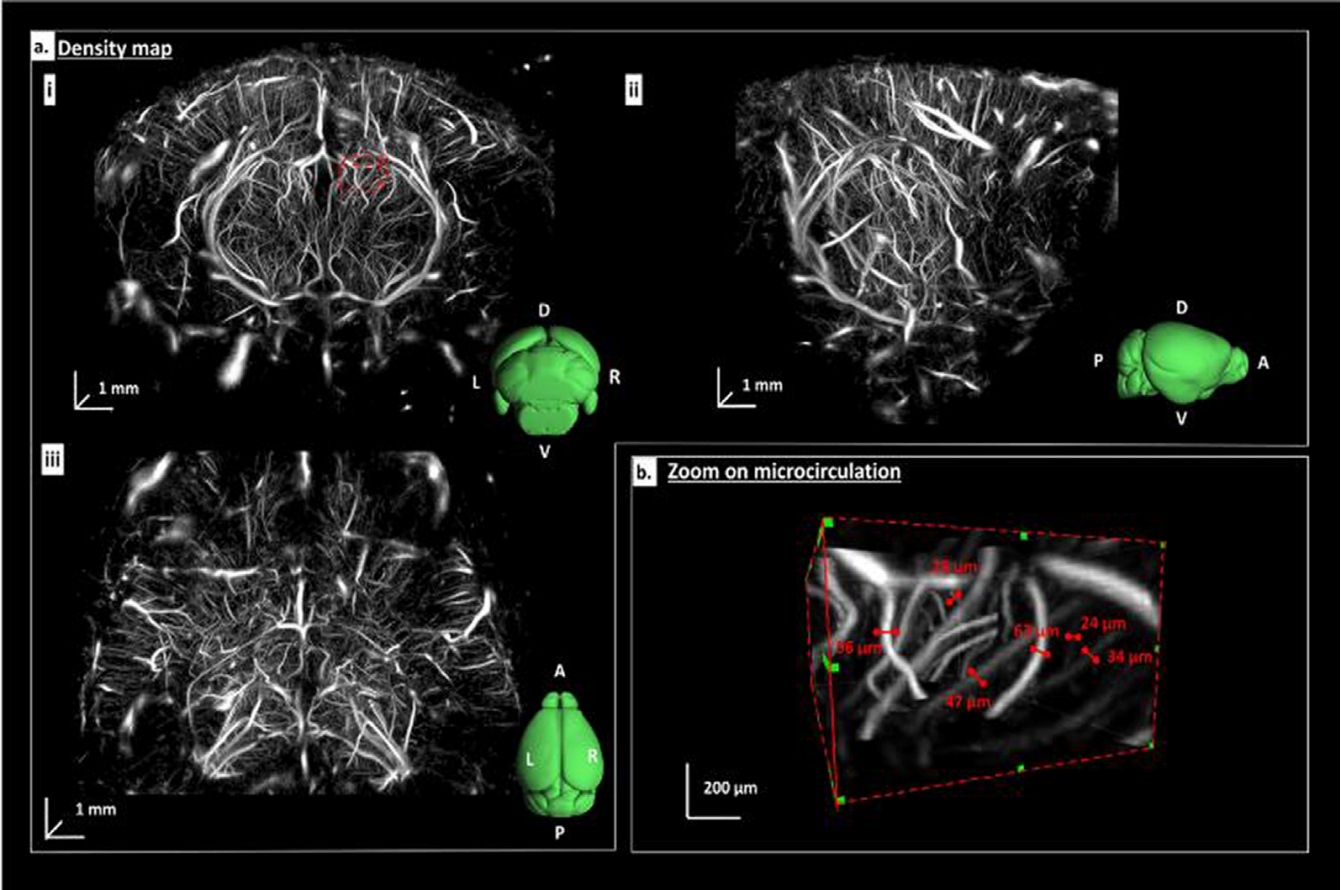
a) 3D ULM of a mouse brain in coronal (i) sagittal (ii) and top (iii) views. b) The zoom shows resolution at the microvascular level with measurements of the vessel diameters. Adapted from [57] under the creative commons license CC-BY-4.0.

First impressive *in-vivo* results were presented by Demeulenaere et al. [57] (see Fig. 6), who were able to record the vasculature of a complete mouse brain at super-resolution with the Vermon matrix probe within 15 minutes. Fig. 6 shows different views of the 3D ULM volume together with the orientation of the mouse brain from the Allen atlas. This study shows the feasibility of achieving super-resolution even with the limitations in focusing and image quality that the Vermon matrix transducer imposes. Similar results from 7.5 minute acquisitions of the rat brain with the same transducer were recently achieved by Chavignion et al. [66]. In both cases no motion compensation was used because of the limited motion for brain imaging. The translation of these 3D results to clinical imaging, e.g., for tumor angiogenesis in breast cancer, will need sophisticated 3D motion compensation and a further reduction in acquisition times. In this respect, a compromise has to be found between the completeness and resolution of the vessel tree and the acquisition times that still enables the characterization of the microvasculature for the specific application.

**Figure 7:**
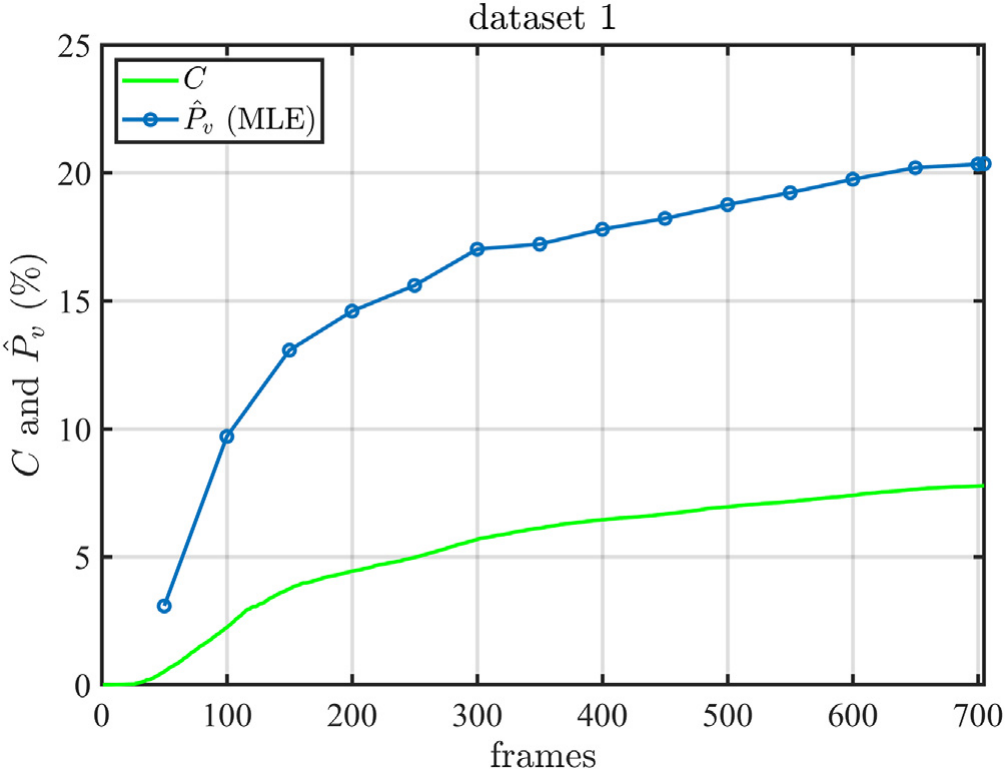
Estimation of the final percentage of pixels containing vessels (${\hat{P}}_{v}$, blue) in dependence of the number of frames used for the estimation. For comparison, the percentage of pixels containing vessels in the current frame (C, green) is shown. Adapted from [22]Fig. 5a showing only the relevant curves under the creative commons license CC-BY-4.0.

## 5 Applications

The application of ULM is of potential interest for pathologies and changes that are related to the morphology or physiology of the microvasculature. This includes pathologies that manifest first in small vessels, particularly angiogenesis related to neoplasms or inflammation, or pathologies that may show a reduction of microvascular flow. Here, the microvasculature is of interest for diagnosis, prediction of therapeutic response, and monitoring of therapy. But also, for the monitoring of angiogenesis related to regeneration or healing processes, ULM could play a role. Additionally, ULM can support the general understanding of organ function at the microvascular level.

### 5.1 Tumors

One of the main foci so far is the application to tumors. It is well known that early detection of malignant tumors, preferably before the formation of metastases, is crucial to increase the chances of successful treatment [70]. The assessment of malignant tumors by ULM is particularly promising because pathological angiogenesis builds vessels distinctly different from normal vasculature. The main differences are the formation of tortuous vessels and a different hierarchical structure, expressed in fewer branching [13], [14]. Accordingly, using ULM a higher tortuosity of microvessels in tumor-bearing tissue (subcutaneous fibrosarcoma) in a rat could be shown by Lin et al. [62]. In [7], we used distances between vessels differentiated by flow velocities to characterize tumors in mice (types A431, MLS, A549). This publication was also the first to clinically visualize the change in microvasculature under chemotherapy. Also using clinical scanners, so far, Kanoulas et al. and Huang et al. reconstructed a prostate tumor [71] and a pancreatic and a breast tumor [72], respectively, the second using a high frame-rate clinical scanner.

Additionally, Yi et al. proposed to use ULM for the evaluation of thermal ablation treatment at liver tumors to differentiate residual tumor from a so-called inflammatory reaction band (IRB) [73]. Acute responses to treatment may cause increased perfusion of the tissue and a corresponding enhanced brightness in CEUS images that is difficult to distinguish from that of a residual tumor [74], [75]. The expected differences in the morphology and function of the microvasculature between residual tumor and IRB could likely be better characterized by ULM.

Because microcirculation variations in lymph nodes can be indicative for metastasis (sentinel nodes), Zhu et al. used ULM to characterize vessel sizes and blood flow velocities as a first step in healthy rabbits [61] and Yan et al. showed that the visualization of a human lymph node using a clinical scanner is possible [50].

### 5.2 Inflammatory processes

In addition to angiogenesis in tumor formation, pathological angiogenesis may also occur in inflammatory processes. For example, rheumatoid arthritis is associated with hypervascularization of the synovium. Whether angiogenesis is the cause or consequence in this disease seems to be still unclear [76]. In contrast, it is known that plaques in arteries (atherosclerosis) cause changes in the vasa vasorum [77], and a correlation between intraplaque neovascularization and cardiovascular events was shown in [78]. In these areas, vasa vasorum imaging with ULM could become an important diagnostic tool.

Moreover, inflammatory response is expected to play a role for the development of acute-on-chronic liver failure [79]. Using ULM, Huang et al. could already show that acute-on-chronic liver failure becomes manifest in a vascular structure distinct from that of a healthy liver [72].

### 5.3 Kidney and heart

A further research focus is on the kidney. For instance, the progression of chronic kidney disease is associated with dysfunction of the microvasculature and a reduction in capillary density [80], [81]. Particularly in the transition of acute to chronic kidney disease, changes in the microvasculature may play a significant role. Foiret et al. already reconstructed the microvasculature of a rat kidney [25], Song et al. of a rabbit kidney [33], Chen et al. of a mouse kidney [53], and Huang et al. of a healthy human kidney [72], demonstrating the feasibility of ULM despite the strong difference in flow velocities present in the kidney.

Particularly challenging seemed the reconstruction of the heart microvasculature because of the strong movements. However, ECG-triggered synchronization of the acquisition with the heart cycle can be used to record data during diastole. This made it possible to reconstruct the 3D coronary microvascular anatomy and flow velocity in isolated beating rat hearts by Demeulenaere et al. [67], a first step towards a differentiated analysis of coronary occlusion and heart attack.

### 5.4 Other pathologies

Other areas of application could be diabetes or neurodegenerative diseases. Gosh et al. [82] could show differences of parameters derived from ULM at skeletal muscles between lean and obese mice after the infusion of insulin/glucose which could be of interest for measuring diabetic dysfunction.

Whether pathologies of small vessels are related or contribute to neurodegenerative diseases like Parkinson or Alzheimer disease is under discussion [83], [84], [85]. Since Lowerison et al. showed a lower blood flow velocity and an increased vascular tortuosity across all brain regions, and a decreased blood volume in the cerebral cortex for aged mice compared to young mice [49], here, ULM could possibly make a contribution as well.

### 5.5 Physiological angiogenesis

Physiological angiogenesis plays a role in injury healing or graft tolerance. Using ULM, Beliard et al. [11] investigated the vascular alterations over time after acute spinal cord injuries in rat-tails showing significant local differences in blood volume, flow velocities, and tortuosity index. The aim of these investigations is to characterize the severity of lesions based on the microvasculature and to be able to predict the prognosis of treatment.

### 5.6 Functional imaging

Finally, ULM can be of interest to better understand the general functioning of tissue and organs, whether pathological or not. For example, functional imaging of the brain to investigate neurovascular coupling [23] and of the placenta to discriminate maternal and fetal blood flow [86] were already realized using ultrafast Doppler US. However, as already discussed, Doppler techniques are limited in resolution, so ULM could be used to reveal more detail and thus make more information accessible. That even the microvascular flow velocity and flow rate of a beating heart under normal conditions and during vasodilator adenosine infusion can be analyzed with ULM [67] shows its great potential.

## 6 Summary

Since its first applications in preclinical imaging of tumor vasculature in mice about one decade ago, ULM has recently taken its first steps to clinical applications. It shows promising diagnostic results for diseases manifesting in the morphology of the microvasculature. One remaining limitation for clinical use are the relatively long acquisition times for a single super-resolution image in the order of 1-15 minutes, because on the one hand the MBs have to be relatively sparse and, on the other hand, each vessel to be imaged must be flowed through by at least one MB. Therefore, localization of single MBs in higher concentrations is an active area of research. In this regard, it also has to be answered by future studies which degree of reconstruction of the vasculature is needed to derive quantitative parameters of the vessel morphology. E.g., sampling only a few microvessels in less than one minute may be sufficient to quantify their degree of tortuosity or to identify general directions of microvascular flow.

Another prerequisite of the method is the necessity to administer contrast agents, which currently have regulatory clearance only for specific applications. However, if relevant clinical questions can be answered by ULM, the spectrum of CEUS applications can be expected to be extended. Alternatively, super-resolution techniques that avoid the use of contrast agents and instead localize the echoes of the erythrocytes were proposed recently [87] and have to be investigated further in comparison with CEUS-based ULM.

Sometimes it is claimed (e.g., [73]), that ULM is not real-time capable because of its time-consuming post-processing. Obviously, no complete reconstruction can be available at the beginning of the measurement since the flow of the MBs has to be observed. However, by now it is possible to carry out the comparatively complex postprocessing as far as possible in parallel with the acquisition process, so that the reconstruction can be available at the end of the recording or shortly after.

While impressive images with nearly complete vascular trees of the brains of mice and rats were recorded within 7.5-15 minutes [57], [66], clinical applications will not allow for such long acquisition times and will exhibit considerably more tissue motion. Though in-plane motion can be compensated in 2D imaging, in daily clinical routine examinations out-of-plane motion will be difficult to avoid. Therefore, ULM has an inherent need for imaging in 3D. The recorded volume has to be large enough to compensate for motion in the region of interest and in some applications volume slices around a 2D plane of interest may suffice and allow for high volume rates. Additionally, MBs must be detected and imaging sequences with nonlinear detection of contrast by harmonic imaging together with grayscale imaging for motion estimation have to be implemented in 3D.

Currently, however, clinical applications must still work with the available acquisition modes and DICOM data, which is suboptimal for ULM and limits the accuracy of the method. Nevertheless, the clinical testing of ULM for various applications is the major research task for the coming years deciding about the future of the method. Once manufacturers start to implement ULM on their systems and develop dedicated imaging modes, the method will unfold its full clinical potential.

## Declaration of Competing Interest

The authors declare that they have no known competing financial interests or personal relationships that could have appeared to influence the work reported in this paper.
